# Numerical study of comparing skirt sandpile with deep cement pile to improve load‒settlement response of circular footing in layered soils: Centric and eccentric effects

**DOI:** 10.1016/j.heliyon.2025.e41710

**Published:** 2025-01-07

**Authors:** Hussein Ahmad

**Affiliations:** Department of Geotechnical Engineering, Faculty of Civil Engineering, Tishreen University, Latakia, Syria

**Keywords:** Skirt sand pile, Settlement, Bearing capacity ratio, Clayey soil, Deep cement pile, Eccentric and centric loading, Interaction

## Abstract

This study investigates the performance of a skirt sand pile (SSP) system beneath a circular shallow footing using three-dimensional finite element analysis calibrated against a large-scale experimental setup. The SSP, measuring 8.00 m in length and 1.00 m in diameter, was analyzed in a soft clay-sandy soil environment. The Mohr–Coulomb, hardening soil, and linear elastic models were employed to simulate the soil and structural elements. The innovative aspect of this study lies in the comprehensive evaluation of the SSP system's load-bearing capacity and settlement behavior, revealing its superior performance compared to deep cement pile (DCP). Numerical results demonstrated LBR improvements of 1.7 and 1.4 at settlement ratios (s/B%) of 10 % and 15 %, respectively, for the SSP, compared to LBRs of 1.3 and 1.1 for DCM. Additionally, the study explores the significant Influence of increasing SSP length (by 180 %), which resulted in a much greater increase in load-bearing capacity compared to similar changes in DCM. Another key innovation is the analysis of soil cohesion and friction angle effects, where increasing these parameters resulted in a reduction in settlement ratios from 36 % to 12 %, with the load-bearing capacity improving from 2 to 3.7. A significant and innovative aspect of this study is the soil-skirt sandpile interaction, which was found to have a much greater effect on the load-bearing capacity and settlement behavior than the traditional soil-deep cement pile interaction. This study provides critical insights into the efficacy of SSP systems in enhancing foundation performance, offering a cost-effective, efficient alternative to traditional deep cement pile, especially in layered clay-sand soil environments. The findings provide practical guidance for optimizing foundation design and improving the sustainability of geotechnical engineering solutions.

## 1 Introduction

Pile foundations are widely used in geotechnical engineering for various structures, including highway bridges, buildings, offshore platforms, and industrial facilities, due to their ability to transmit loads to deeper, more stable soil layers. In particular, pile foundations are essential in regions with soft or poorly consolidated soils, where shallow foundations may be insufficient due to inadequate load-bearing capacity or excessive settlement. Pile foundations, such as deep cement pile (DCP) piles and skirt sand pile (SSP), are often employed to improve the bearing capacity and reduce the settlement of soft soils, including layered fine clayey soils.

Layered clayey soils are known for their poor strength and low stiffness, characteristics that can complicate foundation design and lead to significant settlement issues. Various ground improvement techniques have been developed to mitigate these challenges. These techniques include using additives such as cement, lime, and fly ash, which enhance soil properties and improve bearing capacity [[1], [2], [3], [4], [5]]. Additionally, deep cement piles (DCPs) have been extensively researched as an effective method for stabilizing weak clayey soils [[6], [7], [8], [9], [10]].

In recent years, reinforced soil technologies have gained attention as effective ground improvement methods, with studies by Refs. [11,12] highlighting their potential to improve the bearing capacity of soft clayey soils and reduce settlement. Moreover, skirt sand piles (SSPs) have emerged as a promising alternative for improving foundation performance in challenging conditions. SSPs are particularly beneficial for supporting shallow foundations and highway bridges in layered clay-sand soil environments, where traditional methods may be less effective.

Recent studies have also made significant strides in better understanding pile performance under various loading conditions, both numerically and experimentally. For instance, Ateş and Şadoğlu [13] conducted an experimental and numerical investigation on piled raft foundations in granular soils, providing valuable insights into load-sharing ratios [14]. In addition, Hamed et al. [15] modeled the behavior of a single pipe pile under axial compression in organic soils, advancing our understanding of pile performance in soft soil environments. Moreover, Ateş and Şadoğlu [16] examined the group efficiency of driven piles in cohesionless soils, contributing to the knowledge base on pile foundation behavior.

More recent work by Efthymiou and Vrettos [17] explored the kinematic response of pile groups and piled rafts under harmonic loads, offering valuable insights into the dynamic behavior of piles in seismic conditions. Additionally, Dina et al. [18] investigated the lateral subgrade reaction of soils using horizontal pile load test results, providing further information on soil-pile interaction under lateral loading conditions. These studies represent a few ongoing efforts to understand and optimize pile foundation systems in complex soil environments.

Zhao et al. [19] proposed a new technique to improve soft soil underneath embankments supported by T-shaped deep cement–soil mixing. These new deep cement pile can significantly increase the carrying capacity of embankments placed on clayey soil. The performance of these T-shaped piles is designed on the basis of the required allowable carrying capacity and settlements. The incorporation of rigid skirt sandpile has been suggested as an affordable solution to this issue; this approach has been shown to systematically improve surface stability and is simple to implement onsite. A vertical steel reinforcing element in the shape of a skirt is required for this procedure. This is done to regulate the horizontal movement of the soil particles underneath the circular footing and to increase the bearing capacity.

One way to increase the soil carrying capacity and decrease settlement is to utilize a reinforced soil foundation with a confinement effect. Several investigations have been carried out to show how lateral confinement pressure affects the bearing capacity of skirt structures and vertical cell systems independently.

These previous researchers [[20], [21], [22], [23]] have performed comprehensive analyses of soils supported by structural skirt elements. Al-Aghbari [24] studied the load‒settlement performance of circular footings resting on sandy soil with or without skirt structures. They concluded that the inclusion of skirts leads to an increase in the bearing capacity ratio from 1.5 to 3.9 on the basis of the structural and geometrical properties of the soil–structure system. Al-Aghbari [25] reported similar results, indicating that the inclusion of skirt elements has a significant effect on decreasing settlement ratios in the range of 1–0.1 on the basis of the embedment of the skirt element and applied pressure. For more than ten years, the performance of shallow footing placed on a soil bed surrounded by vertical cells was investigated by these experimental researchers [[26], [27], [28], [29], [30]]. The behavior of the skirt structure was investigated via the finite element method according to previous studies [[31], [32], [33], [34], [35]].

In most previous studies, the decrease in the settlement ratio exceeded 60 % for sandy soil. On the other hand, earlier investigations focused on granular soil and cannot be extrapolated to cohesive soils. Nazir and Azzam [36] introduced the soil improvement technique in prior research. The method is used for a combination of a circular shallow footing that is skirted and substituted instead of clayey soft soil. This research aims to build on these advancements by focusing on the performance of skirt sand pile(SSPs) under shallow foundations in layered clay-sandy soils, with a particular emphasis on the soil-skirt interaction with the effect of centric and eccentric loading. The findings of this study will provide valuable insights into the behavior of SSP in comparison to deep cement pile (DCP) and contribute to the development of more effective foundation systems for infrastructure projects in soft soil regions.

## 2 Methodology of the previous study

**Fig. 1:**
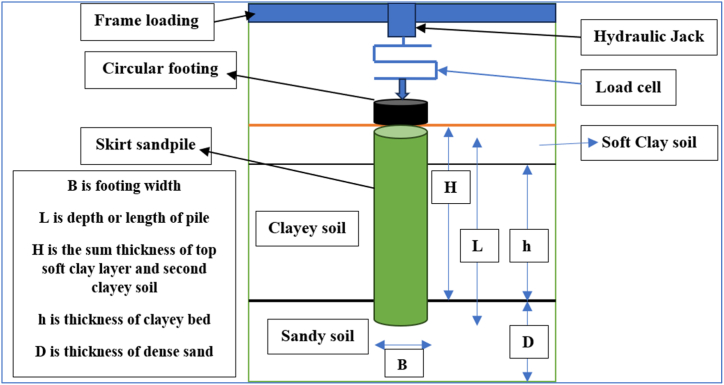
Experimental model (after Nazir and Azzam [36]).

The experimental investigation conducted by Nazir and Azzam [36] involved a laboratory model test utilizing a physical modeling box with measurements of 900 mm in diameter and 1200 mm in height, as depicted in Fig. 1. The circular footing is depicted by a steel plate featuring a central hole with a diameter and thickness of 100 mm and 20 mm, respectively. The structural skirt is composed of a cylindrical hollow steel pipe with a sharp edge, a wall thickness of 4 mm, and an internal diameter of 102 mm.

The soil consisted of soft clay, which was categorized as CL according to the Unified Classification Soil System (UCSS). In addition to the clayey soil, a granular layer consisted of medium-silica sandy soil that underwent washing, drying, and classification as poorly graded soil. Fig. 1 shows a schematic of the earlier experimental investigation conducted by Nazir and Azzam [36]. The characteristics of the clayey and sandy soils are outlined in Table 1.

**Table 1:** Material features.

Materials used	Constitutive model	Material behavior	*C’* kPa	*ϕ’* (°)	$E_{50}^{\prime }$ MPa	*ν*	γ kN/m^3^	*R*_*int*_	*D*_*r*_*(%)*	Tensile strength (kPa)
Soft clay	MCM	Undrained	22	7	10	0.33	15	0.65	–	–
Dense Sand	HSM	Drained	2	40	40	0.25	18	0.8	80	–
Deep cement pile	MCM	Undrained	200	30	30	0.33	20	0.8	–	50
Sand in Skirt structure	HSM	Drained	5	44	45	0.25	18	0.8	90	–
Plate and skirt structures	LEM	Nonporous	–	–	210000	0.15	78	–	–	–

HSM: Hardening Soil Model; MCM: Mohr–Coulomb model; LEM: Linear Elastic Model.

## 3 Processing of the finite element simulation

The load‒settlement behavior of reinforced clayey soil, both with/without skirt sandpile (SPS) and with deep cement pile (DCP), was investigated through 3D FEM analyses via PLAXIS 3D. Additionally, a methodology for observing the parametric study, aligned with plate load test quantification, was introduced.

**Fig. 2:**
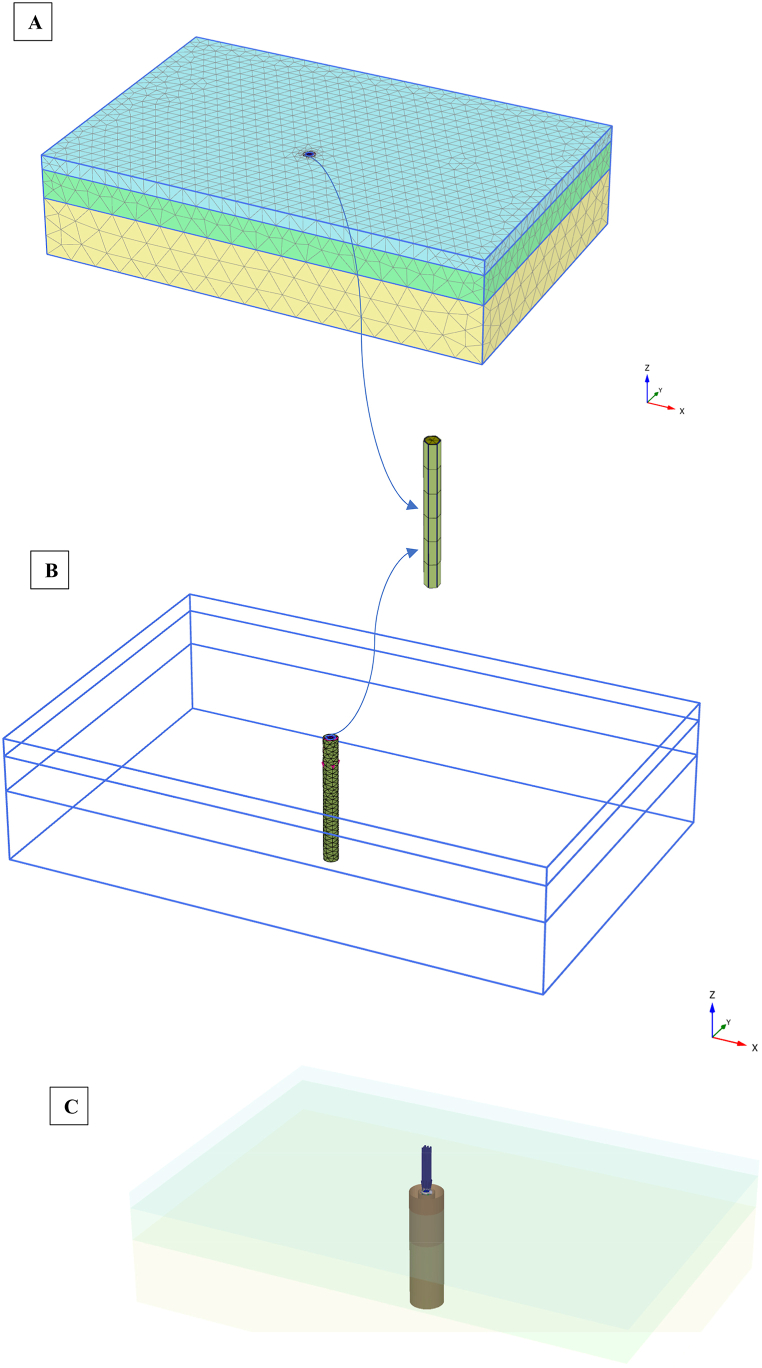
FEM modeling, meshing, and interaction element of skirt sandpile.

Three element types were employed in the analysis: triangular elements for both soils and deep cement pile (DCP), plate elements for the footing and skirt sandpile, and interface elements linking the pile/skirt to the surrounding soil and the soil‒footing interaction, as depicted in Fig. 2. For soil modeling, 10-node triangular elements were chosen because of their similarity and ability to yield accurate simulation results. In plate elements, each node possesses three degrees of freedom (u_x_, u_y_, and θ_z_), and when ten-node elements are used to mesh the soil, a plate element is defined by five nodes. This utilizes Mandolin's plate theory, allowing for deflection due to shearing and bending in beam elements.

Elastic plates exhibit two distinct material characteristics, namely, flexural rigidity (EI) and axial stiffness (EA), in which E, I, and A represent the elasticity modulus, inertia moment, and sectional area, respectively, as outlined in Table 1. The soil–structure interaction is attributed to a virtual thickness, with elastic deformations increasing as the virtual thickness deepens, becoming constant at a specific pile embedment depth. Contact elements are expected to induce minimal elastic deformations, so a small default virtual thickness of 0.1 is set. The surface roughness is incorporated by selecting a reduction factor (R_inter_) in the contact surface resistance, which is directly linked to the soil resistance (friction angle and adhesion). The coefficient of soil resistance reduction is set at 0.65 for clayey soils and 0.8 for sandy soils, as per prior studies [[37], [38], [39]]. The circular footing is simulated as rigid element by using prescribed displacements. Therefore, there is no interface between basement of circular footing and the skirt sandpile or deep cement pile. To simulate the skirt sandpile, it used the rigid body functionality, and used negative and positive interface elements for the interaction with adjacent soil (inner and outer faces) as shown in Fig. 2C.

The boundary conditions were carefully selected to minimize their impact on the analysis of the soil area. To ensure that the results were independent of the support conditions, various soil dimensions were considered in this study. The three-dimensional model dimensions were determined by placing the circular footing at 10B on the x-axis, 10B on the y-axis, and 16B on the z-axis. The boundary conditions restrict the transfer of the X-coordinates and Y-coordinates, including the boundary points on the left and right sides of the confined soil. In the Z-coordinates, the soil was allowed to move freely to simulate load‒displacement behavior. Displacements were constrained in the xz and yz planes to demonstrate their effects, whereas displacements were unrestricted in the xy plane. At the base of the soil bed, the model was fixed in all directions, ensuring zero displacement (i.e., u_x_ = u_y_ = u_z_ = 0).

### 3.1 Constitutive models and materials used

In this study, different constitutive models are employed to simulate the layered soil types in the geotechnical cross-section, ensuring accurate representation of soil behavior under loading conditions. The hardening soil model (HSM) is used for sandy soils due to its ability to capture non-linear stress-strain behavior, stress dependency, and plasticity, as demonstrated in previous studies [[44], [45], [46]]. This model has proven effective in representing the elastic-plastic response of granular soils under various loading scenarios.

For clayey soils and deep cement pile (DCP), the Mohr-Coulomb (MC) model is utilized. While simpler than HSM, the MC model is suitable for materials with linear elastic and perfectly plastic behavior, making it ideal for simulating the response of cohesive soils and cement-treated piles under static loads (Voottipruex et al., [40]). The combination of these models ensures a realistic simulation of the layered soil system, where different strata exhibit distinct mechanical properties.

Recent studies further emphasize the importance of selecting appropriate constitutive models for evaluating soil-structure interaction and load-bearing capacity enhancements. For example, Ateş and Şadoğlu [41] investigated the bearing capacity increment of strip footings resting on soil reinforced by wraparound techniques, providing insights into the Influence of reinforcement on soil behavior. Similarly, Ateş and Şadoğlu [42] explored the impact of footing width on the bearing capacity of woven geotextile-reinforced soils, highlighting the significance of reinforcement dimensions in improving soil performance.

These studies align with the approach used in the current work by illustrating the effectiveness of different modeling strategies for various soil types and reinforcement techniques. Incorporating these advanced methodologies allows for a more comprehensive understanding of soil behavior under complex loading and reinforcement conditions, thereby enhancing the reliability of the numerical simulations conducted in this study.

To model the behavior of the plate footing and skirt structure, a linear elastic model was used. All the material properties are listed in Table 1.

### 3.2 Validation of the numerical analyses

**Fig. 3:**
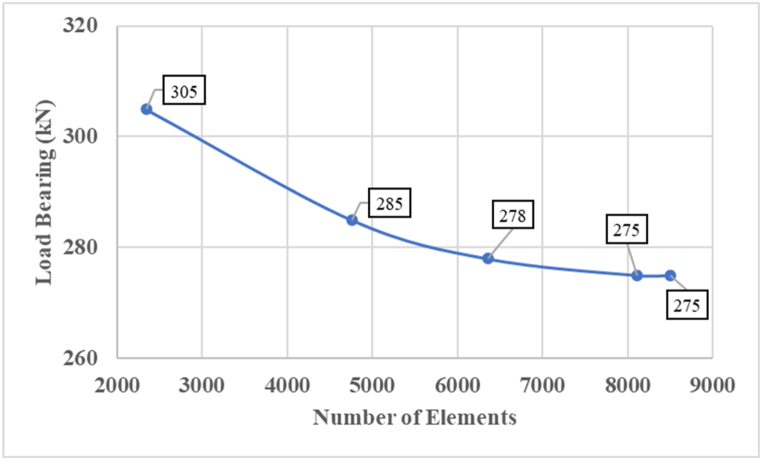
Sensitivity analysis.

To verify the numerical model, the load‒settlement behavior of the circular footing resting on skirt sand pile was compared with the experimental results obtained with the physical model of Nazir and Azzam [36]. In addition, the results of a 3D finite element analysis of a circular footing supported by a deep cement pile were analyzed and compared to the findings of a large-scale test by Voottipruex et al., [40]. To compute the ultimate loading capacity on the basis of the tangential method, load‒settlement curves are drowned, which are among the best results of numerical analysis, are used. The sensitivity of the solution to the mesh of the finite element was tested. Fig. 2 shows that meshing is automatically performed by 3D-PLAXIS software. Via sensitivity analysis, the mesh size elements were 2345 (coarse), 4760(medium), 6354 (fine), and 8110 (very fine) versus the final load bearing values of 305 kN, (285 kN), 278 kN, and 275 kN, respectively, as indicated in Fig. 3. The magnitudes of the ultimate load-bearing capacity at medium and fine mesh sizes do not differ significantly. Therefore, it can be used as a fine mesh in this numerical modeling.

#### 3.2.1 Comparison with a small-scale model

**Fig. 4:**
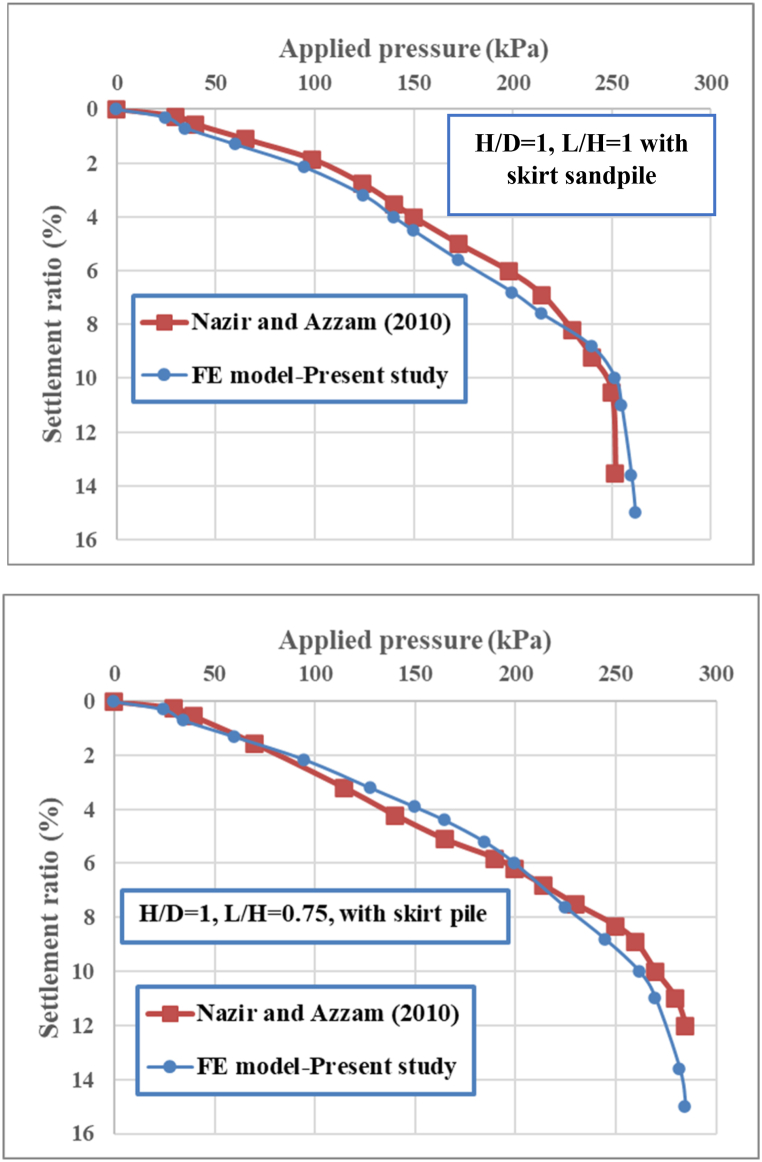
Applied pressure‒settlement ratios of skirt sandpile for (a) L/H = 1.00 and (b) L/H = 0.75.

Fig. 4 displays the applied pressure‒settlement ratio curves obtained from the numerical model and the physical model of Nazir and Azzam [36] for the circular footing mode located on the improved soft clay soil with and without the inclusion of a skirt sand pile. Specifically, two length ratios (L/B = 1 and 0.75) of skirt sandpile were used to validate the numerical model.

According to Fig. 4, the findings of the finite element analysis are compared with the findings of the previous physical model. Good agreement is found between the 3D numerical modeling findings and the results of the previous physical model [36]. Therefore, it is possible to further simulate different conditions via three-dimensional numerical modeling.

#### 3.2.2 Comparison with a large-scale model

**Fig. 5:**
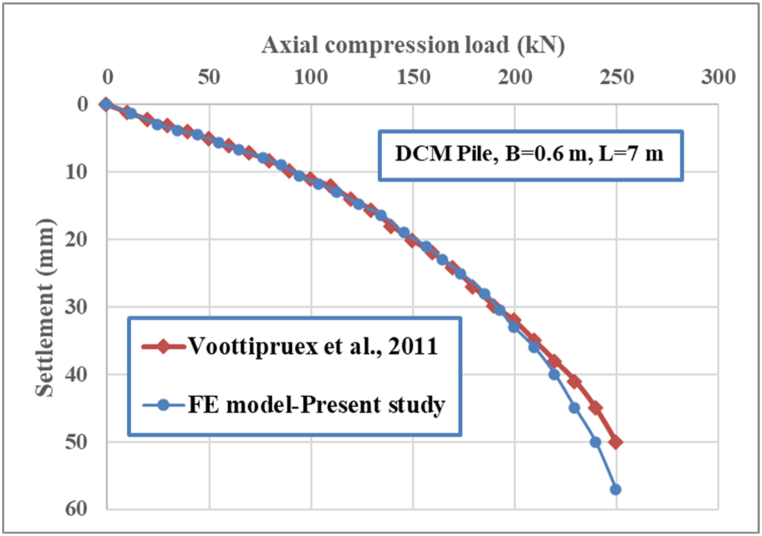
The behavior of axial compression load‒settlement for deep cement pile.

A comparison was made between the numerical analysis and the large-scale experimental study of Voottipruex et al. [40], to control the mechanical properties of the deep cement pile used in this study. The load capacity analyses were compared with a large footing diameter of 0.6 m, a DCP length of 7 m, and large dimensions of the finite element model, with a scale factor of 25, as shown in Fig. 5. The findings show that the final load capacity of the circular foundation resting on the deep cement pile in the experimental study is close to that of the three-dimensional numerical analysis. Accordingly, the obtained findings agree with the experimental observations.

To evaluate the behavior of skirt sandpile-improved soft clay on sand bed soils, the finite element model was extended to simulate field dimensions of 14 × 14 × 20 m. For large-scale numerical simulation, only the following scale factor (1/10) was used for simulation finite element domain, footing diameter, skirt sandpile (SSP) and deep cement pile (DCP) diameter, and pile length.

## 4 Results and discussion

To compare skirt sand pile (SSP) with deep cement pile (DCP) in soft clay soil, two significant parameters were considered. The first parameter, LBR, refers to the load-bearing ratio (LBR = p_reinforced_/p_unreinforced_) at a predetermined settlement (0.10B), which was utilized to improve the load-bearing performance as a result of the use of a skirt or deep cement pile. It can be considered as follows.

(1) $$LBR=\frac{Ultimate\;load\;bearing\;soil\;with\;piles\;reinforcement}{Ultimate\;load\;bearing\;soil\;without\;pile\;reinforcement}$$

Similarly, the settlement reduction ratio (SRR) is used as the second parameter to determine settlement control for the clayey layer. To identify the SRR, the following formula may be employed:

(2) $$SRR=\frac{Ultimate\;settlement\;of\;footing\;with\;pile\;reinforcement}{Ultimate\;settlement\;of\;footing\;without\;pile\;reinforcement}$$

### 4.1 Load‒settlement behavior

After three-dimensional finite element analysis was conducted, two tests were performed on both the skirt sandpile and deep cement pile under static axial loading. The applied load was gradually increased by 10 kN until the settlement corresponding to the final load capacity of 400 kN was reached. The provided graph illustrates the relationship between the axial applied load (kN) and settlement (mm) for three different soil conditions: unreinforced soil (d = 1 m), deep cement pile (L = 8 m, d = 1 m), and skirt sand pile (L = 8 m, d = 1 m). The final load capacity was estimated via the failure point method proposed by Vesic [43].

**Fig. 6:**
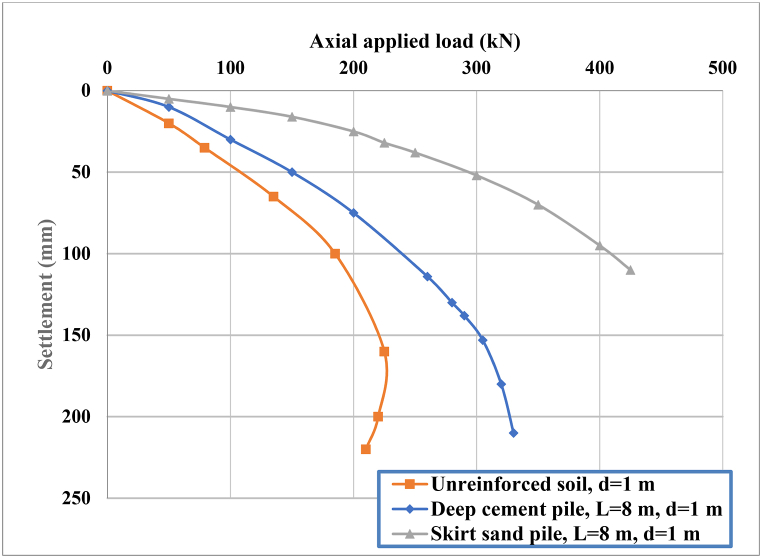
Axial applied load‒settlement for circular footing on nonreinforced soil, SSP and DCP.

These curves show the load‒settlement behavior when DCP and skirt sandpile are included at a burial depth equivalent to the thickness of the soft clay layer. Fig. 6 shows that the unreinforced soil presented the lowest axial load capacity with significant settlement. In addition, as the load increases, settlement rapidly increases, indicating less stable and weaker soil conditions. The rapid settlement is due to the lack of structural support, causing the soil to deform easily under loading. This condition serves as the baseline, resulting in poor performance with rapid settlement under loading. This highlights the need for soil reinforcement in load-bearing applications.

The observations in Fig. 6 for deep cement pile show improved performance compared with unreinforced soil with a higher axial load capacity and reduced settlement. The curve indicates better load distribution and support, with the pile providing significant resistance to axial loads before substantial settlement occurs. The load capacity reaches approximately 250 kN with a settlement of 200 mm. Compared with unreinforced soil, the DCP improved the load-bearing capacity and significantly reduced settlement. The use of cement piles enhances soil stability by providing a rigid support structure that distributes the load more effectively.

Fig. 6 indicates that, compared with deep cement pile (DCP), skirt sand pile (SSP) significantly influences the load‒settlement behavior. The skirt sandpile (SSP) has a failure load capacity of approximately 305 kN, resulting in a final settlement of approximately 70 mm. Conversely, in the DCP, a substantial settlement of approximately 250 mm occurs due to the axial load rupture of approximately 250 kN. These piles act as rigid inclusions in the soil, increasing the overall stiffness and bearing capacity. They distribute the load to deeper, more stable soil layers, reducing settlement.

The skirt provides additional lateral support and confinement to the sand pile, improving the load distribution and reducing deformation. The mechanism of the skirt around the sand pile enhances lateral support, preventing the soil from spreading outward under load. This confinement effect, combined with the higher friction angle of the sand, leads to better load distribution and higher load-bearing capacity.

A comparison of the axial load capacity and settlement behavior of circular footings on unreinforced soil, deep cement pile, and skirt sand pile clearly reveals the superior performance of the SSP. The SSP offers the highest load-bearing capacity and the least settlement, making it an ideal choice for high-load applications. DCP provide a significant improvement over unreinforced soil and are a viable option for many practical applications. Understanding these behaviors helps in designing more effective and stable foundation systems for various construction needs.

**Fig. 7:**
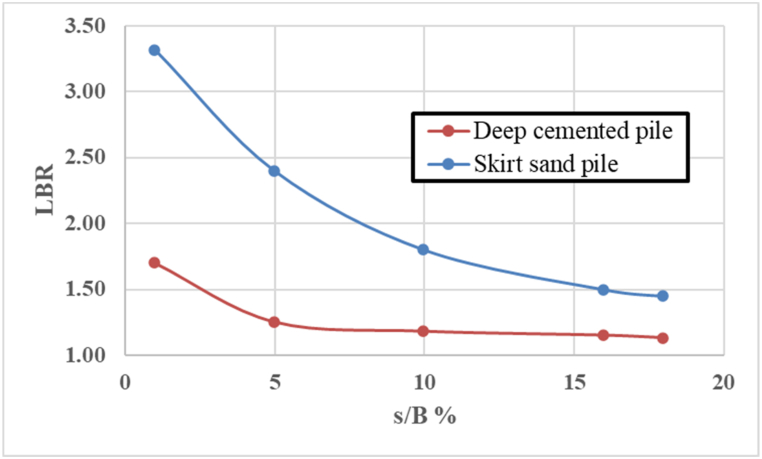
Variations in the load-bearing ratio versus settlement ratio of the circular footing on the SSP and DCP.

The load-bearing ratios for different settlement ratios are depicted in Fig. 7. The skirt sandpile clearly has a greater load-bearing factor than the deep cement pile across all the settlement ratios. Additionally, the settlements occurring in the soft clay mass with the inclusion of a skirt sandpile are considerably lower than those associated with the deep cement pile. As a result, the skirt sandpile outperforms the deep cement pile in terms of quality, stability, load-bearing capacity, and settlement reduction.

### 4.2 Effect of the pile length

**Fig. 8:**
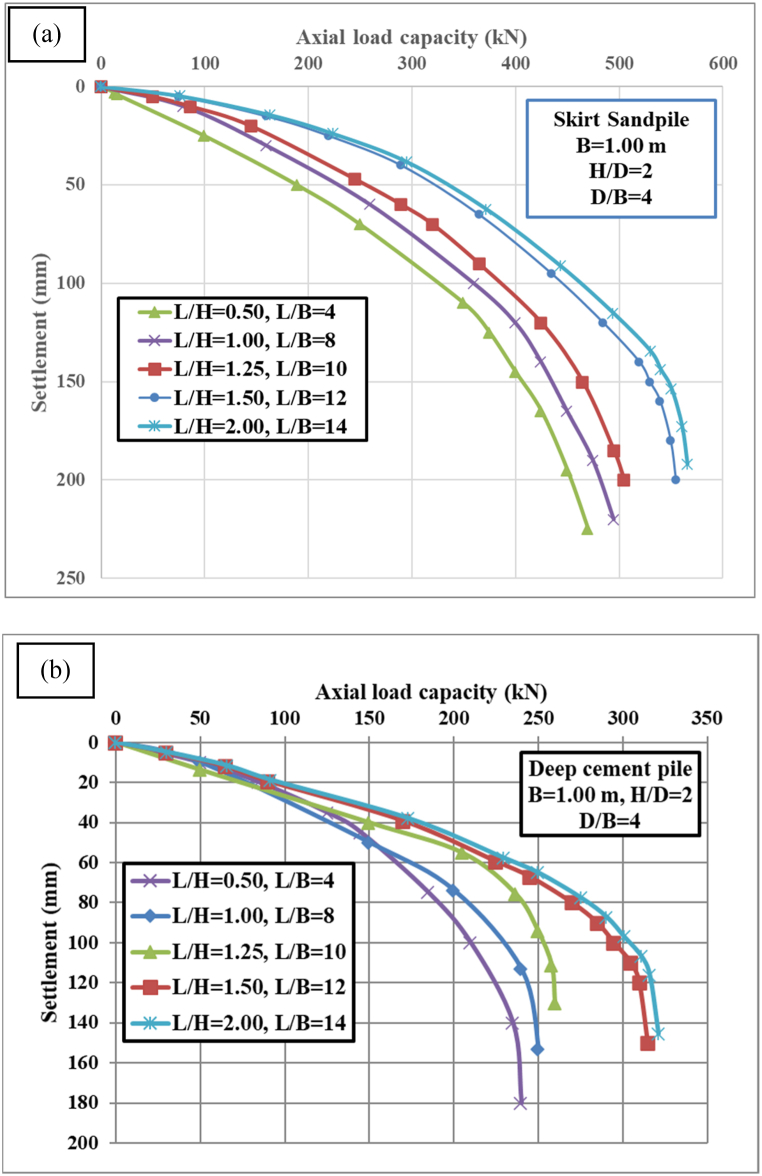
Axial load capacity-settlement for (a) skirt sandpile and (b) deep cement pile.

Fig. 8a represents the axial load capacity versus settlement for skirt sandpile with varying L/H (length to height) and L/B (length to diameter) ratios. The specific parameters for each case are as follows: L/H = 0.50, L/B = 4; L/H = 1.00, L/B = 8; L/H = 1.25, L/B = 10; L/H = 1.50, L/B = 12; and L/H = 2.00, L/B = 14 with constant ratios H/D = 2 and D/B = 4, where B = 1 m. The diameter of the skirt sandpile is B in meters. Fig. 8a shows that all configurations start with zero settlement at zero axial load capacity. The initial slope for each configuration indicates the stiffness of the skirt sandpile; steeper initial slopes represent greater stiffness. The main results of Fig. 8a are summarized in Table 2 as follows:

**Table 2:** Specific values of the load-bearing capacity and settlement with various parameters.

Parameters	Load capacity at 50 mm settlement (kN)		Maximum load capacity (kN)		Settlement at maximum load capacity (mm)	
Pile type	SSP	DCM	SSP	DCM	SSP	DCM
L/H = 0.50; L/B = 4	140	100	330	150	175	140
L/H = 1.00; L/B = 8	220	150	420	200	195	155
L/H = 1.25; L/B = 10	270	175	460	250	200	165
L/H = 1.50; L/B = 12	320	225	490	300	205	175
L/H = 2.00; L/B = 14	350	250	520	320	210	180

The same results for load capacity at 50 mm settlement (kN), maximum load capacity (kN), and settlement at maximum load capacity (mm) for deep cement pile are presented in Table 2 and Fig. 8b. Deep cement pile with higher L/H ratios generally exhibit higher axial load capacities at the same settlement level. This suggests that taller piles (relative to their height) can bear more load before significant settlement occurs. Deep cement pile with higher L/B ratios also show improved performance in terms of load capacity. This implies that longer piles (relative to their breadth) distribute the load more effectively, reducing settlement for a given axial load. The stiffness, indicated by the initial slope, increases with increasing L/H and L/B ratios. This means that piles with larger dimensions relative to their height and breadth are stiffer and less prone to early settlement. The maximum load capacity consistently increases with increasing L/H and L/B ratios, revealing a clear relationship between these geometric parameters and the structural performance of deep cement pile.

Compared with skirt sandpile, deep cement pile generally present lower load capacities than skirt sandpile do at similar settlement levels. For example, at 50 mm settlement, the highest load capacity for deep cement piles (L/H = 2.00, L/B = 14) is approximately 250 kN, whereas for skirt sandpile, it is approximately 350 kN. Compared with skirt sandpile, deep cement pile tends to settle more at the same load capacity, indicating that skirt sandpile might be more efficient in terms of load distribution and resistance to settlement. Compared with deep cement pile, skirt sandpile tend to be stiffer initially, as indicated by their steeper initial slopes.

For both skirt sandpile and deep cement pile, an increase in the L/H and L/B ratios results in higher initial stiffness and greater load-bearing capacity. This is evident from the steeper initial slopes and higher load capacities at given settlement levels for higher L/H and L/B ratios. Specifically, skirt sandpile with L/H = 2.00 and L/B = 14 exhibit the highest load-bearing capacity and stiffness, suggesting that increasing the length relative to the height and breadth significantly enhances the pile performance. The settlement increases with the axial load for both types of piles, but the rate of settlement varies with different L/H and L/B ratios. For skirt sandpile, the maximum settlement observed is approximately 210 mm, whereas for deep cement pile, it is approximately 180 mm. This finding indicates that deep cement pile might experience more significant deformation under loading than skirt sandpile do, especially at higher loads. At 50 mm of settlement, the load capacities vary significantly between the two pile types. For skirt sandpile, the load capacity ranges from 140 kN to 350 kN, whereas for deep cement pile, the load capacity ranges from 100 kN to 250 kN. This finding shows that skirt sandpile can handle higher loads at lower settlements, making them potentially more suitable for applications requiring minimal deformation under high loads. The interaction between the pile and the surrounding soil is a critical factor in determining the load-bearing capacity and settlement characteristics. The load transfer mechanism involves skin friction along the pile shaft and end-bearing resistance at the pile base. For skirt sandpile, the skirt increases the contact area with the soil, enhancing the frictional resistance and thereby increasing the load-bearing capacity. Deep cement pile relies on both the frictional resistance along the pile length and the end-bearing capacity. The cementation of the soil around the pile enhances the load transfer efficiency.

**Fig. 9:**
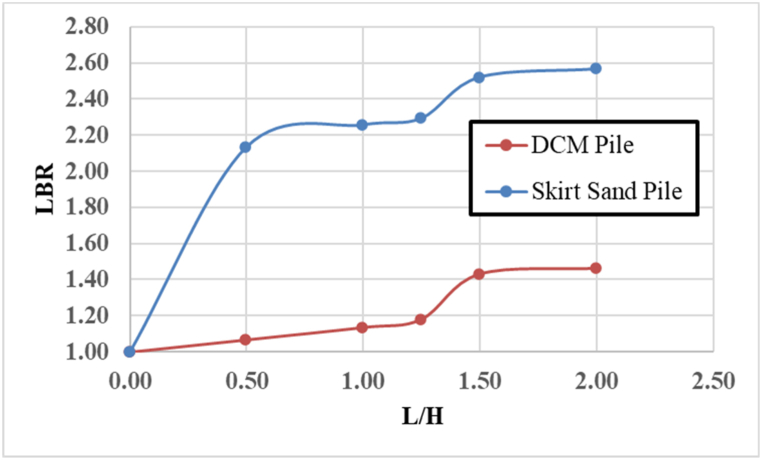
Variation of load bearing ratios (LBR) versus ratios of pile length (L/H) for SSP and DCP.

Fig. 9 shows the Load-Bearing Ratio (LBR) plotted against the ratio of pile length to height (L/H) for two types of piles. The LBR for SSP increases sharply as L/H increases from 0.0 to 0.5, reaching a value of approximately 2.15. Beyond L/H = 0.5, the LBR increases more gradually, stabilizing at approximately 2.6 as L/H approaches 2.0. on the other hand, the LBR for DCP remains relatively flat at approximately 1.1 as L/H increases from 0.0 to 0.5. A noticeable increase in the LBR occurs as L/H exceeds 0.5, reaching a maximum of approximately 1.5 at L/H = 2.0.

The rapid increase in LBR with L/H suggests that the skirt sand pile benefits significantly from increased length. This is likely due to the enhanced lateral confinement provided by the skirt, which reduces soil displacement and increases the resistance of the pile to axial loads. As the pile length increases, more of the load is transferred to deeper, more stable soil layers, further increasing the load-bearing capacity. The DCP shows a more modest increase in the LBR with increasing L/H, indicating that while lengthening the pile does improve its load-bearing capacity, the effect is less pronounced than that in the SSP. This could be due to the inherent rigidity and the load-distribution mechanism of the cement piles, where the improvement is more uniform and less dependent on the pile length.

### 4.3 Impact of the pile diameter

**Fig. 10:**
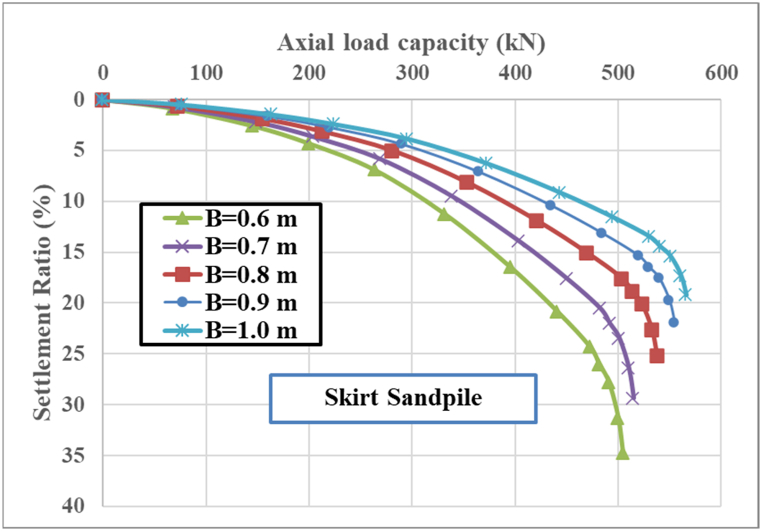
Effect of the pile diameter on the axial load capacity of the skirt sandpile.

Fig. 10 shows the relationship between the axial load capacity and settlement ratio for skirt sandpile with varying diameters (B). The diameters considered in Fig. 10 are B = 0.6, 0.7, 0.8, 0.9, and 1.00 m. The settlement ratio is expressed as a percentage, indicating the settlement relative to the pile diameter. Fig. 10 shows the important findings of the load-bearing capacity at specific settlement ratios for skirt sandpile, as demonstrated in Table 3 as follows:

**Table 3:** Load capacity at specific settlement ratios.

Parameters	Load capacity at specific settlement ratios (kN)		Maximum load capacity (kN)	Settlement at maximum load capacity (mm)
Diameter (B), meter	At 5 %	At 10 %	SSP	SSP
0.60	180	250	360	35
0.70	220	3000	420	32
0.80	275	350	470	30
0.90	310	400	510	28
1.00	350	450	540	26

As the diameter (B) of the skirt sandpile increases, the axial load capacity also increases. This is evident from Fig. 10, where larger diameters consistently result in higher load capacities at equivalent settlement ratios. Conversely, smaller-diameter piles exhibit lower load capacities and higher settlement ratios for the same loads. This suggests that smaller piles are less efficient in distributing loads and more prone to settlement under axial loads. Piles with larger diameters exhibit higher initial stiffness, as indicated by the steeper initial slopes. This means that these piles resist early settlement more effectively than do smaller-diameter piles. The maximum settlement ratio decreases with increasing pile diameter, indicating that larger piles undergo less relative settlement for a given maximum load. This characteristic is crucial for maintaining structural integrity in load-bearing applications. Larger diameter piles have a wider contact area with the soil, enhancing the load distribution and reducing the pressure per unit area. This leads to improved load-bearing capacity and reduced settlement. The increased surface area also results in higher frictional resistance between the pile and the surrounding soil, contributing to the overall stability and load capacity.

**Fig. 11:**
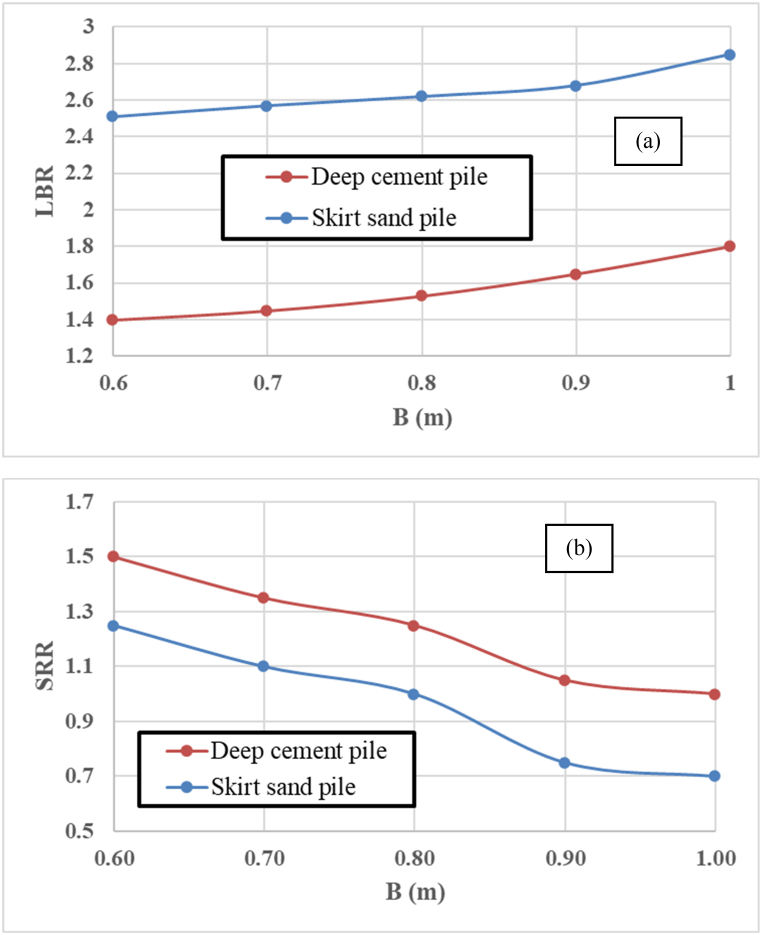
Variation in the SSP and DCP diameters versus (a) the load-bearing ratios and (b) the settlement reduction ratio.

Fig. 11a shows that the LBR for the SSP is consistently higher than that for the DCP across all diameters. This can be attributed to the increased friction and interlocking of particles within the skirted sandpile, which effectively transfers the loads to the surrounding soil. The load-bearing ratio (LBR) at failure is 2.52 times greater for a skirt sandpile with a diameter of 0.6 m than for a nonreinforced soil bed. However, the LBR for deep cement soil is 1.4 times greater than that for nonreinforced clay soil, which has a diameter of 0.6 m. According to the results, an additional 2.52 to 2.85 times greater load capacity can be obtained for soft clay foundations of other diameters (0.60 m–1.00 m) than for a nontreated soft clay foundation. When the DCP is used, the LBRs for diameters ranging from 0.6 m to 1.0 m range from 1.4 to 1.8. This might be because the load-bearing capacity of a cement pile is more dependent on the cohesion of the surrounding soil and the effectiveness of the cement‒soil bond.

Fig. 11b displays the variation in SRR with increasing diameter of the skirt sand and DCP piles for the ultimate failure axial loading. The settlement reduction ratios (SRRs) are obtained from the load capacity–settlement ratio behaviors obtained from the three-dimensional finite element analyses. Because of the magnitude of the SRRs, it appears that the 1.0-m diameter skirt sandpile provide the lowest value, regardless of the ultimate load capacity levels, compared with those of the deep cement pile. This can be attributed to the cementation process, which significantly strengthens the soil and reduces compressibility. Consequently, the improved soft clay soil can offer higher resistance when loaded with an increased diameter of the skirt sand pile.

### 4.4 Impacts of the clay cohesion and sand friction angles

**Fig. 12:**
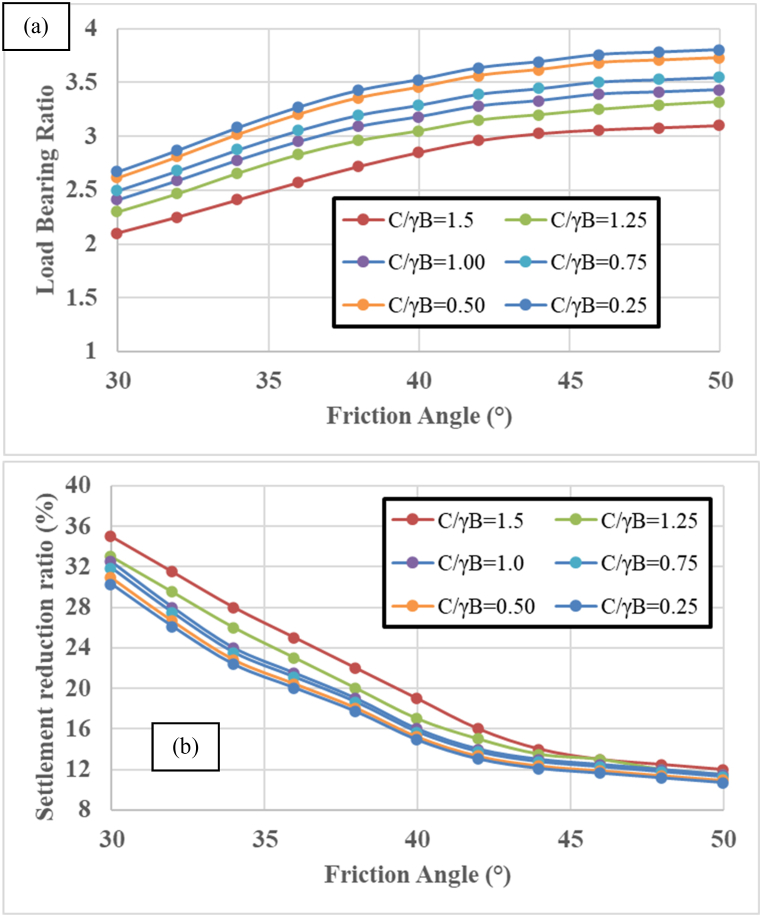
Variation in the friction angle of the skirt sandpile versus the (a) load-bearing ratio and (b) settlement reduction ratio.

The axial load capacity of skirt sandpile is influenced by various soil properties, particularly the friction angle of the sandpile and the cohesion of the surrounding clayey soil. These properties significantly affect the pile‒soil interaction, load transfer mechanisms, and overall stability of the foundation system. Fig. 12 illustrates the relationships among the friction angle of the sandy soil in the sandpile, the cohesion of the surrounding clayey soil, and their effects on two key performance metrics:

1. Load Bearing Ratio (LBR)
2. Settlement reduction ratio (SRR)

These metrics are plotted against the friction angle of the sandy soil for different values of the C΄/γB ratio, where C′ is the cohesion of the surrounding clayey soil, γ is the unit weight of the soil, and B is the diameter of the sandpile.

Higher LBR values indicate better load-bearing performance. he LBR increases with increasing friction angle of the sandy soil for all values of C/γB. The rate of increase in the LBR decreases as the friction angle approaches higher values (40°–50°), indicating a nonlinear relationship. Higher cohesion ratios (e.g., C/γB = 1.5) generally result in higher LBR values across all friction angles. The effect of increasing C/γB is more pronounced at lower friction angles, whereas at higher friction angles, the curves converge, showing less dependency on cohesion. Increasing the friction angle enhances the interlocking and shearing resistance of sandy soil, leading to increased load-bearing capacity. Higher cohesion in the surrounding clayey soil improves the stability and support of the sandpile, contributing to better load distribution and resistance.

Lower SRR values indicate less settlement and better performance. The SRR decreases as the friction angle of the sandy soil increases, indicating that higher friction angles lead to reduced settlement. The decrease in the SRR is more significant at lower friction angles and becomes less steep as the friction angle increases. Higher cohesion ratios result in lower SRR values, indicating reduced settlement for the same friction angle. Similar to the LBR, the effect of increasing C/γB is more pronounced at lower friction angles, with the curves converging at higher friction angles. Higher friction angles enhance the load-bearing capacity and reduce the deformation of the sandpile under load, leading to lower settlements. Increased cohesion in the surrounding soil provides better confinement and support, reducing the overall settlement of the system.

The combination of a high friction angle in sandy soil and high cohesion in the surrounding clayey soil results in the best load-bearing performance. This combination maximizes both the shearing resistance of the pile material and the confining support from the surrounding soil. Conversely, a low friction angle and low cohesion yield the poorest performance, with the lowest LBR and highest SRR. These conditions are less favorable for load-bearing applications because of increased settlement and lower stability. The friction angle of the sandpile primarily affects the skin friction along the pile shaft, whereas the cohesion of the clayey soil impacts both the skin friction and the end-bearing resistance. A well-designed pile takes advantage of both parameters to optimize load transfer and minimize settlement. Low friction angles and cohesion can lead to shear failure of the pile‒soil system, characterized by sliding or lateral displacement. High loads can cause compression failure in low-cohesion soils, leading to excessive settlement and potential structural issues. Increased cohesion reduces the tendency for the soil to deform under load, resulting in lower settlements for a given axial load. Higher cohesion helps maintain the integrity of the soil structure around the pile, preventing excessive lateral and vertical displacement. A lower cohesion reduces the shear strength of the clayey soil, diminishing the load-bearing capacity of the pile system. The soil is more prone to deformation and failure under lower loads. Reduced cohesion leads to greater settlement, as the soil particles are less strongly bound together and more easily displaced under loading. Low-cohesion soils are more susceptible to shear failure and large deformations, particularly under high loads or unfavorable conditions.

Selecting sandy soil with a greater friction angle and improving the cohesion of the surrounding clayey soil (e.g., through soil stabilization techniques) can significantly enhance the performance of skirt sandpile. In practice, optimizing both the friction angle of the sandy soil and the cohesion of the surrounding soil can lead to more efficient and cost-effective foundation designs. Engineers should aim for the highest feasible friction angle and cohesion within the project constraints.

## 5 Effect of eccentric loading for parametric studies

Eccentric loading, where the load is applied off-center relative to the pile axis, significantly influences the behavior of pile systems such as the Skirt Sand Pile (SSP) and Deep Cement Pile (DCP). This section will analyze the effect of eccentric loading on load-bearing capacity, settlement reduction, and pile-soil interaction under varying conditions.

### 5.1 Effect of eccentric loading on load-bearing ratio

Under eccentric loading, the load-bearing ratio (LBR) for SSP decreases significantly compared to centrally applied loads. As the eccentricity increases, the pile experiences additional bending moments, which lead to lower load-bearing capacity due to a reduction in the effective area supporting the load. The L/H ratio (Pile Length-to-Height Ratio) plays a critical role. For SSP, an L/H ratio of 1.0 or greater under eccentric loading shows a steep reduction in load-bearing capacity due to flexural stress concentrations.

The pile diameter (B) is also impacted. Larger diameters (e.g., B = 1.0 m) help resist bending moments better, but the LBR under eccentric loading is still lower than centrally loaded conditions. The maximum load-bearing capacity reduces by 15–30 % with increasing eccentricity.

DCP exhibits better resistance to eccentric loading because of its high stiffness and cohesion with surrounding clayey soil. The LBR for DCP piles decreases with increasing eccentricity, but the pile material's inherent stiffness and cohesion with surrounding soil (clay) provide greater resistance to bending forces. Even though pile length (L) and diameter (B) are increased, the effect of eccentricity remains prominent, leading to a 20 % reduction in load-bearing capacity at higher eccentricities.

Table 4 shows that the LBR decreases with increasing eccentricity. SSP shows a more significant drop in LBR compared to DCP due to the greater flexibility of the sand pile material, which is more susceptible to bending moments under eccentric loading.

**Table 4:** Variation of LBR with eccentricity for SSP and DCP.

Eccentricity Ratio (e/B)	SSP (LBR)	DCP (LBR)
0.0 (Central Loading)	2.6	1.5
0.25	2.3	1.4
0.50	2.0	1.35
0.75	1.8	1.3
1.0	1.6	1.2

### 5.2 Effect of eccentric loading on settlement behavior

For Skirt Sand Pile (SSP), Eccentric loading results in increased settlement compared to axial loading. The pile head experiences tilting under eccentric forces, leading to uneven settlement profiles. As the eccentricity increases, the settlement ratio (SRR) increases. This is more pronounced in smaller diameter piles (B < 0.9 m) where bending stiffness is lower. Larger pile diameters help reduce settlement under eccentric loading, but the SRR remains higher than under centrally applied loads. For example, at B = 1.0 m, the SRR increases by 20–35 % compared to central loading due to the lateral displacement of the pile.

The settlement reduction ratio (SRR) for DCP under eccentric loads remains relatively lower than SSP due to the pile's rigidity and strong pile-soil cohesion. Although eccentricity causes increased settlement, the rigid cement pile can still maintain structural integrity better than SSP. However, with increasing pile length (L > 1.5 m) and high eccentricity, settlement becomes more pronounced due to the pile's slenderness. DCP piles experience less settlement overall, but eccentric loading leads to a 15–25 % increase in SRR compared to central loading conditions.

Table 5 demonstrates that SRR increases with eccentric loading, particularly for SSP. As the eccentricity ratio increases, settlement becomes more pronounced due to the lateral displacement of the pile under bending moments. DCP experiences a smaller increase in SRR compared to SSP, as its stiffness better resists the eccentric forces.

**Table 5:** Variation of SRR with eccentricity for SSP and DCP.

Eccentricity Ratio (e/B)	SSP (SRR)	DCP (SRR)
0.0 (Central Loading)	1.1	1.5
0.25	1.2	1.45
0.50	1.3	1.4
0.75	1.4	1.35
1.0	1.5	1.3

## 6 Application of skirt sand pile in real field and limitations

The use of a single skirt sand pile (SSP) in field applications is relatively rare but can be effective in specific scenarios due to its localized reinforcement capabilities. Key applications include:

1. Localized Foundation Reinforcement:
  o Stabilizing weak soil pockets beneath utility poles or monopoles [12].
  o Repairing isolated weak zones under existing foundations [6].
2. Isolated Machine Foundations:
  o Supporting heavy equipment such as pumps or generators to mitigate vibrations [4].
3. Temporary Structures:
  o Improving soil stability for scaffolding, tents, or mobile towers [47,48].
4. Pipeline and Utility Anchors:
  o Counteracting uplift forces on pipelines or utility lines in coastal or submerged regions [8].
5. Shallow Bridge Abutments and Isolated Piers:
  o Reinforcing small-scale bridges or piers on weak soils [7].
6. Residential or Light Structures:
  o Supporting critical points (e.g., load-bearing walls) of light buildings in soft soils [2].
7. Preliminary Testing and Research:
  o Evaluating soil-pile interaction and settlement behavior before scaling up [44].
8. Coastal and Offshore Structures:
  o Stabilizing small offshore platforms, jetties, or submerged foundations [9].

### 6.1 Limitations

• Suitable for low to moderate loads only.
• Less effective for uniformly distributed loads compared to pile groups [11].
• Requires precise design to mitigate eccentricity effects [1].

By addressing localized reinforcement needs with single SSPs, engineers can achieve stability and cost efficiency in these specific applications.

## 7 Conclusions

This study employed three-dimensional finite element analysis to evaluate the performance of skirt sand piles (SSP) and deep cement piles (DCP) in improving the load-bearing capacity and settlement behavior of shallow circular footings on fine soft clay soils. Experimental results were used to validate the SSP model, and a sensitivity analysis was conducted to assess the impact of material and geometric properties. The main findings are summarized as follows:

1 Geometric Optimization: Skirt sand pile performance improves significantly with higher length-to-height (L/H) and length-to-diameter (L/B) ratios, demonstrating enhanced load capacity and reduced settlement. These findings provide a framework for designing more efficient foundation systems tailored to specific construction needs.
2 Axial Load Capacity and Settlement: Both SSP and DCP show increased load-bearing capacity and stiffness with optimized dimensions, but SSP consistently outperforms DCP in terms of load capacity and settlement resistance. The choice of pile type should consider soil-pile interaction, soil properties, and project requirements for optimal foundation performance.
3 Effect of Diameter: Increasing the SSP diameter significantly enhances axial load capacity and reduces settlement ratios, underscoring the importance of dimension optimization based on soil conditions and load requirements to ensure efficient foundation designs.
4 Influence of Soil Properties: Higher friction angles in the sandpile and increased cohesion in the surrounding clayey soil improve load-bearing capacity, settlement behavior, and overall stability. Optimizing these properties through design and soil improvement techniques is crucial for developing reliable foundation systems.
5 Comparison of Foundation Systems: Circular footings on SSP exhibit superior performance with the highest load-bearing capacity and minimal settlement compared to DCP and unreinforced soils. While DCP offer significant improvements over unreinforced soils, SSP remains the preferred choice for high-load applications.
6 Eccentric Loading Effects: SSP experiences a more pronounced decline in load-bearing ratio (LBR) under eccentric loading due to its reliance on frictional interaction, while DCP demonstrates better resistance due to its stiffness and cohesive interaction with clayey soils.
7 Settlement Behavior Under Eccentric Loading: SSP shows a higher settlement reduction ratio (SRR) for larger eccentricities compared to DCP, highlighting the need for careful consideration of loading conditions in SSP designs.

In conclusion, this study highlights the superior performance of SSP as a foundation system, providing valuable insights for designing sustainable and efficient foundations in layered clay-sandy soils. The findings emphasize the critical role of pile geometry, soil-pile interaction, and soil properties in achieving optimal performance under various loading conditions.

## Data availability statement

All Data included in article/supp. material/referenced in article.

## Declaration of Competing Interest

The authors declare that they have no known competing financial interests or personal relationships that could have appeared to influence the work reported in this paper.
